# Microstructure, Micro-Mechanical and Tribocorrosion Behavior of Oxygen Hardened Ti–13Nb–13Zr Alloy

**DOI:** 10.3390/ma14082088

**Published:** 2021-04-20

**Authors:** Alicja Łukaszczyk, Sławomir Zimowski, Wojciech Pawlak, Beata Dubiel, Tomasz Moskalewicz

**Affiliations:** 1 Faculty of Foundry Engineering, AGH University of Science and Technology, Reymonta 23, 30-059 Kraków, Poland; 2 Faculty of Mechanical Engineering and Robotics, AGH University of Science and Technology, Mickiewicza Av. 30, 30-059 Kraków, Poland; 3 Institute of Materials Science and Technology, Lodz University of Technology, Stefanowskiego 1/15, 90-924 Łódź, Poland; Wojciech.pawlak@p.lodz.pl; 4 Faculty of Metals Engineering and Industrial Computer Science, AGH University of Science and Technology, Czarnowiejska 66, 30-054 Kraków, Poland; bdubiel@agh.edu.pl (B.D.); tmoskale@agh.edu.pl (T.M.)

**Keywords:** oxygen hardening, Ti–13Nb–13Zr alloy, microstructure, tribocorrosion behavior, micromechanical properties

## Abstract

In the present work, an oxygen hardening of near-β phase Ti–13Nb–13Zr alloy in plasma glow discharge at 700–1000 °C was studied. The influence of the surface treatment on the alloy microstructure, tribological and micromechanical properties, and corrosion resistance is presented. A strong influence of the treatment on the hardened zone thickness, refinement of the α’ laths and grain size of the bulk alloy were found. The outer hardened zone contained mainly an oxygen-rich Ti α’ (O) solid solution. The microhardness and elastic modulus of the hardened zone decreased with increasing hardening temperature. The hardened zone thickness, size of the α’ laths, and grain size of the bulk alloy increased with increasing treatment temperature. The wear resistance of the alloy oxygen-hardened at 1000 °C was about two hundred times, and at 700 °C, even five hundred times greater than that of the base alloy. Oxygen hardening also slightly improved the corrosion resistance. Tribocorrosion tests revealed that the alloy hardened at 700 °C was wear-resistant in a corrosive environment, and when the friction process was completed, the passive film was quickly restored. The results show that glow discharge plasma oxidation is a simple and effective method to enhance the micromechanical and tribological performance of the Ti–13Nb–13Zr alloy.

## 1. Introduction

β-phase titanium alloys are characterized by high mechanical strength, good corrosion resistance, high biocompatibility and relatively low Young’s modulus [1,2,3,4,5,6]. In addition, references [7,8] show that Nb and Zr are valuable alloying elements. Nb and Zr have an advantage over possibly toxic elements in body fluid, such as aluminum and vanadium, as they do not generate allergic reactions or genetic changes [9]. Consequently, β or near-β titanium alloys, such as Ti–13Nb–13Zr, have been developed for biological applications [1,10,11].

β-phase titanium alloys are referred to as second-generation titanium biomaterials. One of the rationales behind their development is to address the stress shielding associated with the high Young’s modulus of the implant [2,3,4,5,6]. A lower elastic modulus is best suited for a biomaterial implant, as discussed by different authors [12,13,14]. Mohan and coworkers [15] indicated that the percentage of β phase greatly affects the mechanical properties of the Ti–Mo and Ti–Mo–Zr alloy systems. They showed that Mo improved the β phase stability when the percentage of Mo was increased from 12% to 15%. It was further noted that the elastic modulus of the alloys was lower compared to Ti–6Al–4V and commercially pure Ti. The Ti–15Mo–6Zr alloy had the lowest elastic modulus, thereby confirming that the higher percentage of β phase is the main reason for the lower elastic modulus observed. However, the main drawback of all titanium alloys is their poor tribological performance, as demonstrated by the high coefficient of friction (COF) combined with a tendency for scuffing and adhesive wear [2,3]. Moreover, the friction of titanium alloys in a corrosive environment increases their wear rate [16,17], which is disadvantageous in their application as orthopedic bioimplants. Surface treatment is usually applied to enhance the properties of titanium alloys as bioimplants. Several techniques, including oxidizing [18], nitriding [19], ion implantation [20] and physical vapour deposition (PVD) coating [21], selective laser melting [22], Ar arc melting [12], spark plasma sintering [23], anodization [14], mechanical blending process [15] have been investigated. It is known that oxygen hardening can increase the hardness and tribocorrosion resistance [24,25,26,27,28,29,30].

The characterization of the oxygen diffusion zone and the impact of this zone on macroscopic properties are still subjects of intensive investigation. Dong and Li [28] reported that the boost diffusion oxidation (BDO) process was an effective way to harden a titanium surface. The BDO process involved two steps. In the first step, titanium samples were thermally oxidized in the air. During the second step, the air was removed from the reaction chamber, and pre-oxidized samples were further diffusion treated in a vacuum. The method was successfully employed by Zabler and coworkers [30] for the formation of oxygen diffusion hardening (ODH) zones in the commercially pure Ti (grade 2) and Ti–6Al–4V alloy. A well-controlled hardness and hardened zone depth were achieved. A disadvantage of the process is the formation of an oxide scale on the alloy surface. Thicker titanium oxide layers tend to deteriorate the alloy properties. Jamesh et al. [31] reported that the thermal oxidation process of commercially pure Ti above 850 °C led to oxide scale spallation. This behavior is dangerous in frictional contact, where the detached hard titanium oxide particles accelerate the abrasion.

Januszewicz and Siniarski [32] showed that the assistance of plasma glow discharge during oxidation is of great benefit as it prevents the formation of brittle rutile phase on the treated alloy surface by mechanically damaging the oxide scale by high-energy ions. In our previous work [33], we showed that diffusion hardening of the near-surface zone by interstitial oxygen atoms with using glow discharge plasma in Ar + O_2_ atmosphere is a very effective method to improve the mechanical and tribological properties of two-phase (α + β) Ti–6Al–4V titanium alloy. It was found that the treatment increased the surface hardness about three times, from 3.4 GPa to 10.6 GPa. The wear resistance in dry sliding contact with the Al_2_O_3_ ball was also significantly improved.

The oxygen diffusion hardening of the near-β Ti–13Nb–13Zr was performed by thermal oxidation in an oxygen-containing atmosphere [34]. The results suggest that zirconium plays a key role in the effective oxygen diffusion hardening at 500 °C for alloys of the Ti–Nb-Zr system. It was found that a sufficient Zr concentration (>6 wt.%) permits oxygen diffusion into the alloys. The near-surface hardness appears to increase further as Nb content increases. The high hardness of oxygen hardened Ti–13Nb–13Zr alloy is a result of an outer oxide layer composed of mixed metal (i.e., Ti, Nb, Zr) oxides on top of interstitial oxygen hardened alloy. This dense oxide layer is not only highly passive from a chemical/corrosion point of view but also resistant to abrasive wear, which is attributed to the mechanical stability of the oxide/substrate interface.

To our best knowledge, there are no data on the influence of oxygen plasma glow-discharge on the hardening of the Ti–13Nb–13Zr alloy and the influence of the surface treatment on microstructure and tribological properties in corrosive environments. Therefore, detailed studies on the effect of oxygen plasma glow discharge on the alloy usage properties are necessary. The friction wear corrosion results in metal degradation due to the simultaneous action of mechanical wear and (electro)chemical oxidation. The two mechanisms do not proceed separately and depend on each other in a complex way. In many cases, corrosion is accelerated by wear, and similarly, wear may be affected by corrosion phenomena. The nature of the passive film and the electrochemical conditions play a significant role in friction, wear and corrosion mechanism [35]. When the implants are to be placed in bone tissue, bone ingrowth into porous implant surfaces starts and osteoblast cell adhesion on the substrate increases, which may improve osseointegration. Improved osseointegration provides mechanical stability by interlocking the surrounding bone tissue with the implant. Tribocorrosion is a material degradation process and involves chemical and/or electrochemical interactions between material and its environment during a tribological process. Friction wear corrosion is a degradation process resulting from the combined action of small movements between contacting parts and the environment’s corrosivity [36].

The main purpose of this work is to investigate the effect of oxygen plasma glow discharge temperature on the microstructure development and micromechanical properties of the near-β Ti–13Nb–13Zr alloy. An influence of the applied surface treatment on tribocorrosion behavior of the alloy in Ringer’s solution is also studied.

## 2. Materials and Methods

**Figure 1:**
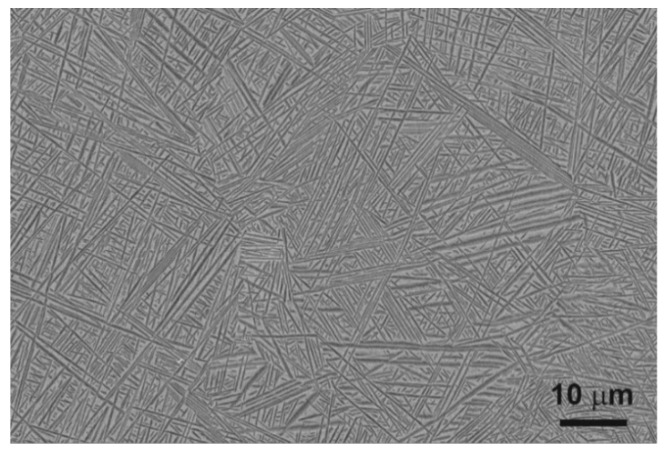
Microstructure of the as-received alloy.

A near-β phase titanium alloy with a chemical composition of 13.6 Nb, 13.6 Zr, 0.06 Fe, 0.04 C, 0.01–0.02 N, 0.001 H, 0.11 O (in wt.%), provided by Xi’an Saite Metal Materials Development Co., Ltd., Xi’an, China was used for oxygen hardening. The β transformation temperature of this alloy is 735 °C [37]. The samples were cut from the bar (27 mm diameter) into discs with a thickness of 1–2 mm. The samples were ground with sandpaper up to 3000-grit until a flat surface was obtained. Next, they were pre-polished with Al_2_O_3_ suspension (grain size 1 µm) and finally polished with silica suspension (0.04 μm) until a mirror finish. The prepared alloy had a fine acicular martensitic morphology composed of α′ (hexagonal close-packed; hcp) and some α″ (orthorhombic, Cmcm space group) laths in β (body-centered cubic; bcc) grains (Figure 1). A detailed description of the microstructure of the as-received alloy has been presented in our previous works [24,25]. The grain size, estimated by image analysis, was in the range of 20–80 μm.

The samples were placed in a quartz tube furnace. After air evacuation, argon of 99.999% purity was purged into the tube. The pressure was adjusted to 10 Pa by flowing argon (flow rate of 30 standard cm^3^/min). When the samples reached the desired temperature of oxidation, i.e., 700 °C or 850 °C or 1000 °C, a glow discharge was started. The glow discharge was generated at 1250 V and an electric current of 45 mA. During discharge, high purity oxygen (99.999%) was mixed with argon and pumped to the furnace in 20 cycles of 1 min duration (flow rate of 6 standard cm^3^/min). During oxygen pumping, the pressure was increased to 12 Pa. After oxidation, cooling to room temperature with working Ar glow discharge was provided.

The microstructure of the oxygen hardened alloy was characterized by light microscopy (LM, ZEISS Axio Imager M1m microscope, Oberkochen, Germany), scanning electron microscopy (SEM, FEI Nova NanoSEM 450 microscope, (Eindhoven, The Netherlands) and transmission electron microscopy (TEM, JEOL JEM-2010 ARP microscope, (Tokyo, Japan) techniques. The phase constitution was studied by selected area electron diffraction (SAED). The Java Electron Microscopy Software (JEMS, version 4.4131U2016, Pierre Stadelmann, Switzerland) was used to interpret the SAED patterns. The lamella from the cross-section of the coated alloy was prepared for the TEM investigation. The samples were thinned using FEI Quanta 3D 200i scanning electron microscope equipped with a Ga+ ion gun, Pt precursor gas injection systems (GIS) and OmniProbe micromanipulator for in situ lift-out [38]. Ion beam accelerating voltage of 30 kV and ion currents in the range of 15 nA–30 pA were applied. The lamella was transferred via a micromanipulator to a TEM half ring, where a focused ion beam (FIB) milling to electron transparency was performed (ion currents of 500–30 pA). FIB deposition process from Pt precursor was used to attach the manipulator probe to the lamella and attach it to the grid. The phase identification was supplemented by energy-dispersive X-ray spectroscopy (SEM-EDS, TEM-EDS) microanalysis and STEM-EDS line analysis.

The open-circuit potential (OCP), linear sweep voltamperometry and electrochemical impedance spectroscopy (EIS) were carried out using a potentiostat Autolab PGSTAT302N (Metrohm Autolab B.V., Utrecht, The Nederlands). The reference electrode was a saturated calomel electrode (SCE), and a platinum plate was used as the counter electrode. Ringer’s solution was used as the electrolyte for the corrosion study. The chemical composition of the Ringer’s solution was: 8.6 g/L NaCl, 0.3 g/L KCl, 0.25 g/L CaCl_2_. Measurements were performed at pH 7.4 in deaerated solutions at 37 °C. The polarization test was performed at a scan rate of 1 mV/s from −1.3 V to +2.2 V vs. SCE. For the EIS measurements, the amplitude was 10 mV; the frequency was 10^5^ Hz to 10^−3^ Hz. The EIS measurements were performed at the OCP potential. The EIS data were fitted using ZView software.

The hardness and elastic modulus of the as-received and oxygen hardened Ti–13Nb–13Zr alloys were determined by micro-combitester (CSM Instruments). The indentation tests were performed using a Vickers diamond indenter. The indenter was loaded with a force of 200 mN and kept for 5 s before un-loading. An analysis of the mechanical properties of the alloy was carried out using the loading/unloading curve by Oliver and Pharr method [39]. It allowed for the determination of the elastic modulus (E_IT_) and hardness (H_IT_). For each sample, ten consecutive experiments at randomly selected places were performed, and the average of 10 measurements was calculated.

The abrasive wear resistance and the friction coefficient of the as-received and treated alloy were determined by friction tests based on the ISO 20808 standard [40]. The tests were carried out in dry sliding contact with an Al_2_O_3_ ball (6 mm diameter) using a ball-on-disc tribotester (ITeE Radom, Radom, Poland). In a typical ball-on-disc arrangement, a counter-element in the form of a ball was pressed against a rotating disc (sample) made of titanium alloy. The tribological tests were repeated three times, with the same parameters: ball load F_n_ = 5 N, sliding speed v = 0.07 m/s, sliding distance s = 1000 m, room temperature 23 °C and relative humidity 55%. The wear rate W_v_ = V/F_n_ × s was determined as the ratio of the volume of material removed during friction (V) to the load (F_n_) and the sliding distance (s). The volume was calculated based on the size of the cross-sectional area of the wear groove. The groove profile was measured with a stylus profilometer in six places around the wear track.

**Figure 2:**
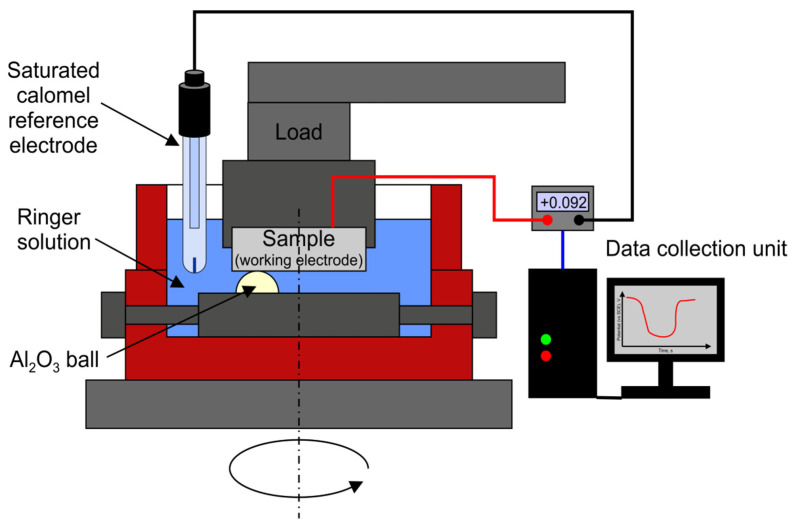
Schematic of the tribological device with parallel corrosion potential measurement during wear tests of the titanium alloy in sliding contact with a ball.

Friction-wear tests with simultaneous measurement of corrosion potential during friction in Ringer’s solution were also performed. To record the changes in the corrosion potential, a 2-electrode system with the working electrode (titanium alloy) and reference electrode (calomel electrode in 3 M KCl) was designed. The schematic of the tribocorrosion device is shown in Figure 2. A specially designed system ensuring an efficient and stable electric contact with the alloy sample was used. In this system, the ball with the holder was moving, whereas the titanium alloy sample was stationary. To fix the ball and sample positions, special polymer holders were used. The same measurement parameters were applied as in the case of dry friction tests. The friction process began after a stable corrosion potential for the titanium alloy was reached. When the friction was activated, a change in the potential was recorded as a result of the wear of the alloys’ surface layer.

Before each test, the alumina ball and titanium alloy were ultrasonically cleaned in ethanol and left to dry. Additionally, the ball used in the tribocorrosion test was also washed in Ringer’s solution. The holders were subjected to an identical cleaning procedure. The whole measurement took place in a plastic vessel filled with Ringer’s solution.

## 3. Results

### 3.1. Microstructure of the Oxygen Hardened Alloy

**Figure 3:**
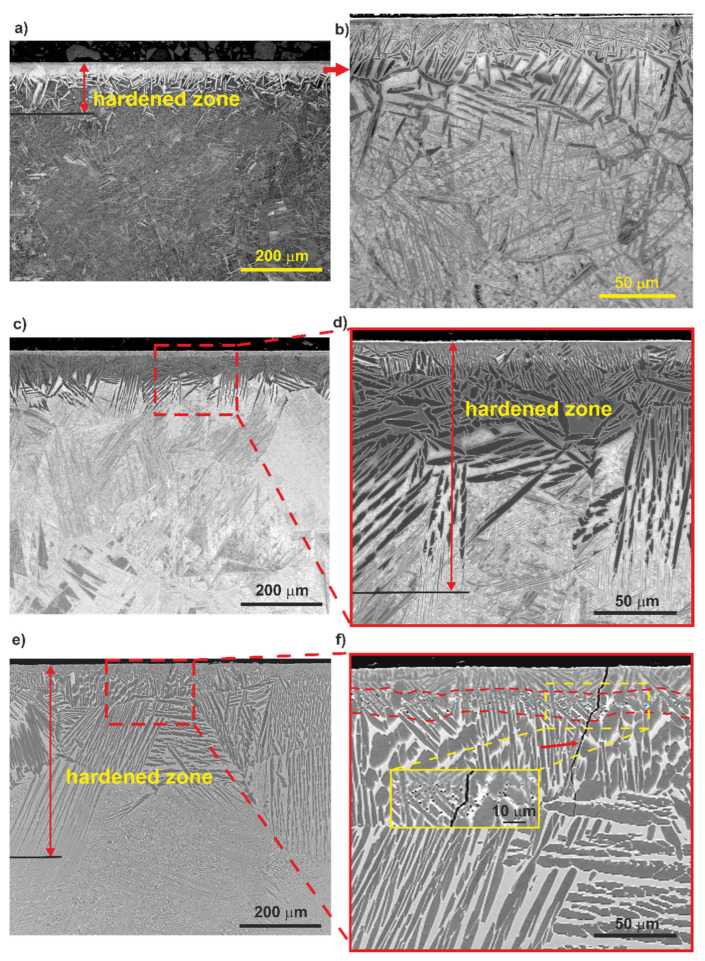
Microstructure of the Ti–13Nb–13Zr alloy after oxygen hardening at 700 °C (**a**,**b**), 850 °C (**c**,**d**) and 1000 °C (**e**,**f**). LM (**a**) and SEM (**b**–**f**), cross-section samples. The zone with voids is marked with a dashed line in Figure 3f. An arrow in Figure 3f indicates a microcrack developed in the near-surface zone.

LM and SEM images of the alloy treated at 700 °C, 850 °C and 1000 °C are shown in Figure 3. The alloy microstructure was dependent on the hardening temperature. The differences in the thickness of the oxygen-rich hardened zone and the grain size of the bulk of the material were observed. The highest thickness of the hardened zone, up to 470 µm, was found in the sample treated at 1000 °C (Figure 3e,f). In the case of alloy treated at 850 °C, the hardened zone thickness was about 160 µm (Figure 3c,d), while in the alloy treated at 700 °C it had the lowest thickness, up to 120 µm (Figure 3a,b). A coarsening of the α’ laths in the oxygen-hardened zone with increasing treatment temperature was also observed. The voids in the hardened zone of the sample treated at 1000 °C were formed at a distance of ~20–30 µm from the surface. It should be noted that the presence of pores usually contributes to the formation of microcracks, which were also sporadically observed in this sample. A typical microcrack is marked with an arrow in Figure 3f. The observed microstructural defects exclude the sample hardened at 1000 °C from the intended biological applications.

Investigation of the cross-section showed a significant grain growth resulting from the hardening process. The grain size estimated from LM and SEM images was in the range of 40–100 µm, 100–250 µm and up to 700 µm for the alloys treated at 700 °C, 850 °C and 1000 °C, respectively.

**Figure 4:**
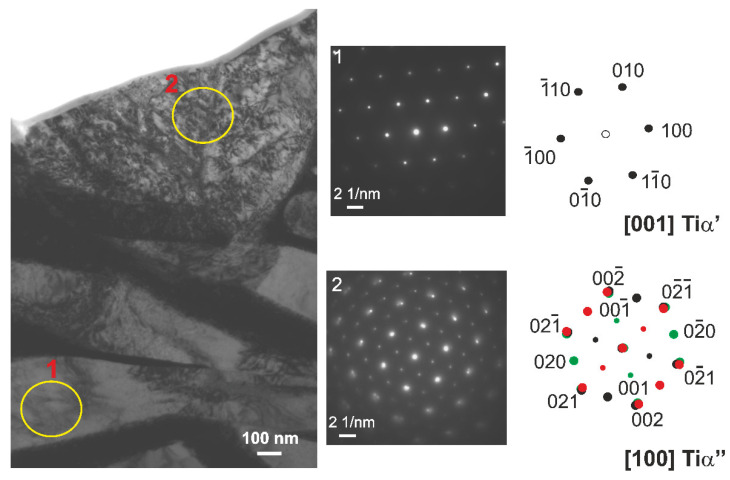
TEM image of the near-surface region in the Ti–13Nb–13Zr alloy after oxygen hardening at 700 °C. SAED patterns of α’ (hcp) and α’’ (orthorhombic, Cmcm) were taken from areas marked with 1 and 2, respectively. In the SAED pattern no. 2, the spots belonging to the three [100] α‘‘ zone axes are marked with black, green and red color. Indices of green spots are given.

**Figure 5:**
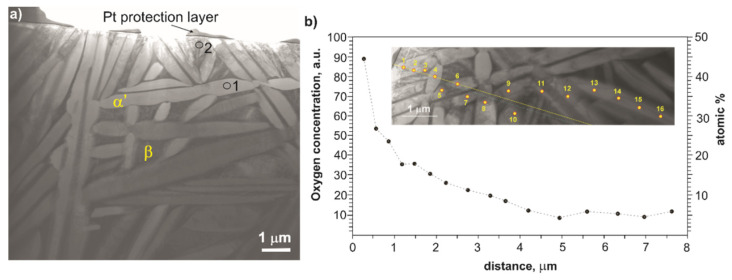
STEM image of the microstructure of the near-surface region in the Ti–13Nb–13Zr alloy cross-section after oxygen hardening at a temperature of 700 °C (**a**) and concentration profile of oxygen obtained by TEM-EDS microanalysis performed in points 1–16 (**b**). The exemplary grains of the α‘ and β phases, as well as the areas 1 and 2 given in Figure 4, are marked in Figure 5a.

The sample treated at 700 °C was selected for a detailed microstructure characterization by TEM and STEM. The TEM and STEM images are shown in Figure 4 and Figure 5. In the near-surface region (depth up to 10 µm from the surface), a high fraction of the Ti α’ (O) solid solution and low amount of fine laths of the Ti α’’ (O) solid solution in β phase were found (Figure 4). In the SAED pattern no. 2, the diffraction spots from crystal planes belonging to particular three [100] Tiα‘‘ zone axes corresponding to three sets of the Ti α’’ laths inclined by the angle of 60° are marked with black, green and red color, respectively.

To examine the concentration profile of oxygen in the near-surface region, a TEM-EDS microanalysis was performed at 16 points located in the α‘ phase at a distance from 0.3 µm to 8 µm. The oxygen concentration profile is presented in Figure 5b. The results confirmed the increased content of oxygen in the zone close to the surface. At a point located 0.3 µm from the outer surface edge of the lamellae, the oxygen concentration was about 47 at.%, and with the increasing distance to 6 μm, it dropped gradually to about 6 at.%. At greater depths in the sample, the oxygen concentration in the α‘ phase remained constant at ~6 at.%. The obtained results of the oxygen distribution should be treated as approximate values since EDS microanalysis gives only a rough indication of light elements concentration. Nevertheless, the result is an indication that the Ti α’ (O) solid solution is enriched in oxygen in the near-surface zone at a depth of up to 6 μm.

In our previous study [33], we showed that the near-surface region of the oxygen diffusion hardened two-phase (α + β) Ti–6Al–4V alloy consisted of Ti α (O) solid solution enriched with oxygen mainly. Oxygen is a strong interstitial solid solution strengthening element of titanium [36]. It is an α phase stabilizer and has a high solubility in the hcp α phase, up to 31.9 at.%. The solubility of oxygen in the β phase is much lower, maximum 8 at.% [41]. Therefore, the presence of the Ti α’ (O) phase in the near-surface zone is preferred due to the diffusion of interstitial oxygen atoms.

According to [39], the plasma glow discharge strengthens the oxygen diffusion into the metallic substrate. It is likely due to an increase in the number of point defects formed during the first stage of the process. In addition, the plasma glow discharge inhibits the formation of the rutile layer on the titanium alloy surface. The oxides formed on the alloy surface act as limited reservoirs of oxygen atoms, which are then forced to diffuse into the alloy matrix and form a solid solution [32]. Therefore, the surface of the alloy investigated in this work was not covered by titanium oxide.

### 3.2. Micromechanical and Tribological Properties

Table 1 shows the micromechanical and tribological properties of the as-received and oxygen hardened Ti–13Nb–13Zr alloy. The surface treatment of the alloy resulted in a significant increase in hardness and elastic modulus than the as-received alloy. Two effects can explain this strengthening: (i) the crystallographic strains generated by the strong lattice deformation that expand the c/a ratio, and (ii) the long-range ordering of the interstitial atoms in the hcp-structure of the host [36]. However, based on the present investigation results, it was found that both the hardness and elastic modulus decreased significantly in the surface layer of hardened alloy with increasing hardening temperature (Table 1). The tendency to lower the hardness with increasing the titanium alloy’s treatment temperature was also noticed elsewhere [42]. The Ti–13Nb–13Zr alloy hardened at 700 °C had the highest microhardness and modulus of elasticity, 12.8 GPa and 180 GPa, respectively. The microhardness of the alloy treated at 1000 °C reached only 6.9 GPa. This behavior is related to the microstructure of the hardened alloy surface layer, particularly to the refinement of the α’ laths at 700 °C. The largest grain size was found in the oxygen-enriched layer of the titanium alloy hardened at 1000 °C. The large grain size does not favor the material strengthening. Additionally, for the alloy treated at 1000 °C, the formation of voids in the hardened zone, located ~20–30 μm from the surface, was observed (Figure 3f). Such microstructure features facilitate a plastic deformation during indentation and result in lower hardness.

The hardening improved the mechanical properties of the Ti–13Nb–13Zr alloy, and both the hardness and elastic modulus increased about three times compared to the baseline alloy. Interstitial oxygen diffusion hardening of the alloy carried out at 700 °C allowed to achieve a hardness comparable to the hardness of the alloy treated by plasma electrolytic oxidation (PEO) in an electrolyte with and without the addition of zirconia nanoparticles [43].

**Figure 6:**
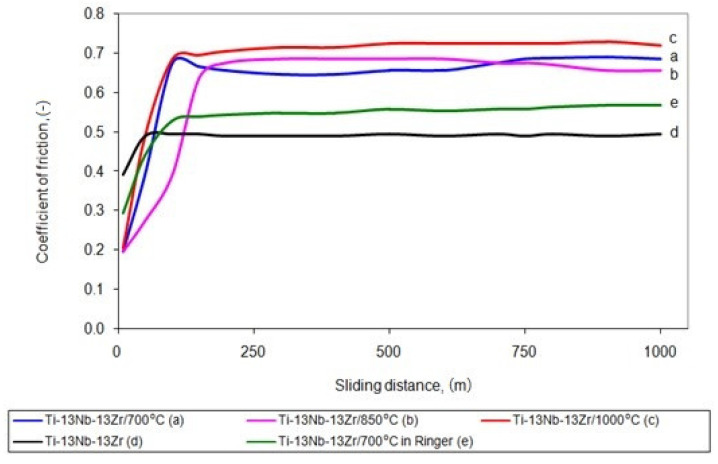
COF of the alloy oxygen hardened at 700 °C (**a**), 850 °C (**b**) and 1000 °C (**c**) compared to as-received alloy (**d**) in dry friction condition as well as the alloy hardened at 700 °C in Ringer’s solution (**e**) against alumina ball.

The wear resistance of the titanium alloy was tested in dry sliding contact. Figure 6 shows the average COF of the as-received and hardened alloy. The COF of the hardened alloy samples in dry sliding contact with the Al_2_O_3_ ball was in the range of 0.63–0.78. A significantly lower COF = 0.50 occurred during the friction of the baseline titanium alloy, and the cooperation with the Al_2_O_3_ counterpart was more stable.

The unstrengthened titanium alloy showed a more significant deformation in the sliding point contact than the hardened alloy since their moduli of elasticity differed significantly. Less deformation should reduce the mechanical component of the friction force and thus the friction coefficient for the hardened alloy. However, such an effect has not been observed as the surface interactions in the friction microcontact had the primary influence on the resistance to motion. The hardened surface layer of the titanium alloy constitutes difficult cooperation conditions in a non-lubricated contact with the hard ceramic Al_2_O_3_ ball. A microstructural investigation has shown that the surface of the alloy examined in this work was not covered by titanium oxide. TiO_2_ could reduce the resistance to motion. The improved friction and wear properties can be attributed to the low-friction TiO_2_ rutile layer [44]. Moreover, the mean contact pressure (p_m_) in the initial period of friction test of the 700 °C hardened alloy was 0.82 GPa, i.e., much higher than the untreated alloy (0.54 GPa). As a result of the sliding interaction of such hard oxide materials, so-called severe friction developed [45], which caused a high resistance to motion. The obtained results are characteristic for this type of material during dry friction [46].

**Figure 7:**
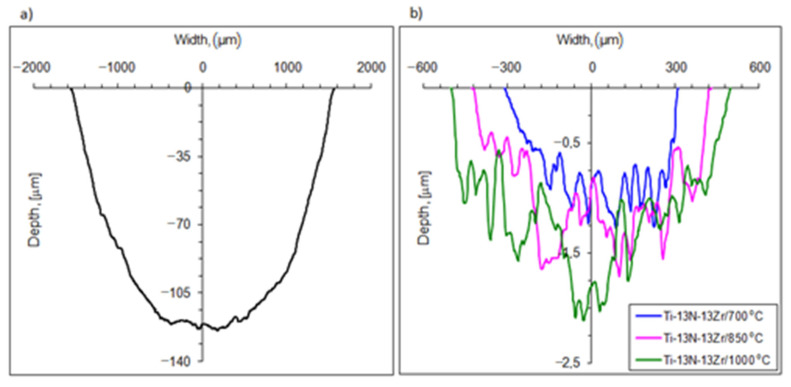
Cross-section profile of wear track of as-received Ti–13Nb–13Zr alloy (**a**) and hardened Ti–13Nb–13Zr alloy (**b**) after dry friction.

The wear resistance of the oxygen-hardened alloy was strongly dependent on the treatment temperature and was at least two hundred times greater than that of the base alloy (Figure 7). The wear rate of the as-received Ti–13Nb–13Zr alloy reached the value of 1250 × 10^−6^ mm^3^/Nm. In comparison, the wear rate for the alloy hardened at 700 °C, 850 °C, and 1000 °C was 2.3 × 10^−6^ mm^3^/Nm, 3.1 × 10^−6^ mm^3^/Nm and 5.8 × 10^−6^ mm^3^/Nm, respectively. Based on the microscopic analysis of the wear track surface, an abrasive wear nature of the hardened titanium alloy and abrasive-adhesive wear of the as-received alloy were found. The wear process of the Ti–13Nb–13Zr alloy is typical of dry friction in contact with a hard counterpart and has already been analyzed in detail elsewhere [47].

**Figure 8:**
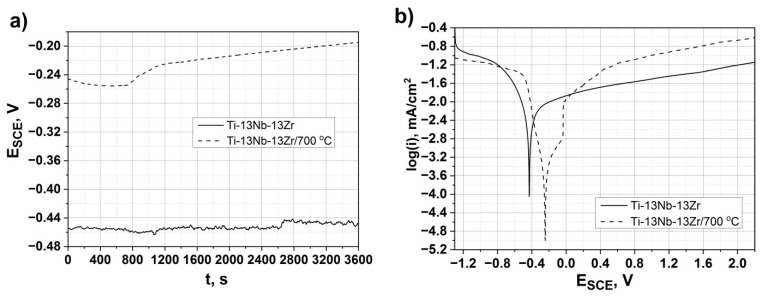
Electrochemical measurements of as-received and oxygen hardened alloy (Ti–13Nb–13Zr/700 °C) in Ringer’s solution at 37 °C, (**a**) evolution of the corrosion potential vs. time and (**b**) polarization curves at 1 mV/s.

The best mechanical and tribological properties were found for the Ti–13Nb–13Zr alloy hardened at 700 °C. Therefore, this alloy was selected for further corrosion resistance tests. Figure 8a shows the evolution of the OCP for the as-received and the alloy hardened at 700 °C. The results of E_ocp_ show that the as-received alloy has a less noble potential than the alloy heat-treated at 700 °C, indicating that the as-received alloy is more susceptible to corrosion. The OCP slightly increased for the hardened alloy and reached a stable value after about 2000s.

The potentiodynamic polarization testing was conducted to understand the corrosion properties of the untreated and treated alloy (Ti–13Nb–13Zr/700 °C) (Figure 8b). Because of an absence of linear regions, the Tafel extrapolation was not applicable to interpret the electrochemical response. In such cases, the corrosion rate could be defined by the limiting current density, which passes through the passivating film, thus becoming a measure of the film protective performance [48]. The passive current density (i_p_) was reduced from 68 µA/cm^2^ for the treated alloy to 20 µA/cm^2^ for the as-received alloy, while the cathodic–anodic transition increased from about −0.43 V up to −0.25 V. These results indicate that the as-received alloy has a slightly smaller corrosion rate than the treated one.

**Figure 9:**
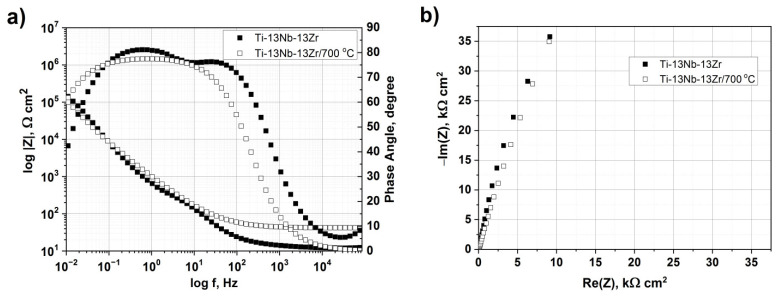
Electrochemical impedance curves of the as-received and oxygen hardened alloy in Ringer’s solution. (**a**) Bode impedance and phase angle plot, (**b**) Nyquist impedance plot.

**Figure 10:**
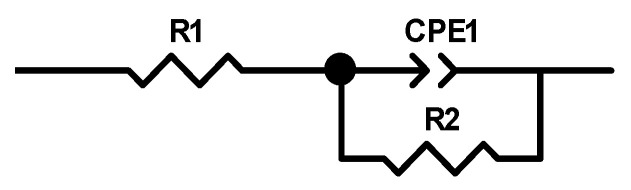
Equivalent circuit used for fitting EIS data.

Figure 9 shows the EIS graphs presented as a Bode plot (Figure 9a) and a Nyquist plot (Figure 9b) of the as-received and hardened alloy in the Ringer’s solution. From Figure 9a, the Z modulus at a lower frequency in the Bode impedance plot indicated a comparable corrosion resistance of the investigated samples. The as-received and treated alloys showed a highly capacitive behavior from medium to low frequencies. The equivalent circuit, as shown in Figure 10, was used to fit the EIS data. According to the double-layer model for the oxygen hardened alloy, the equivalent circuit consisted of the electrolyte resistance (R1), the treated resistance (R2) and the constant phase elements (CPE). A good fitting between the experimental and simulated results was achieved, and the parameters are listed in Table 2.

**Figure 11:**
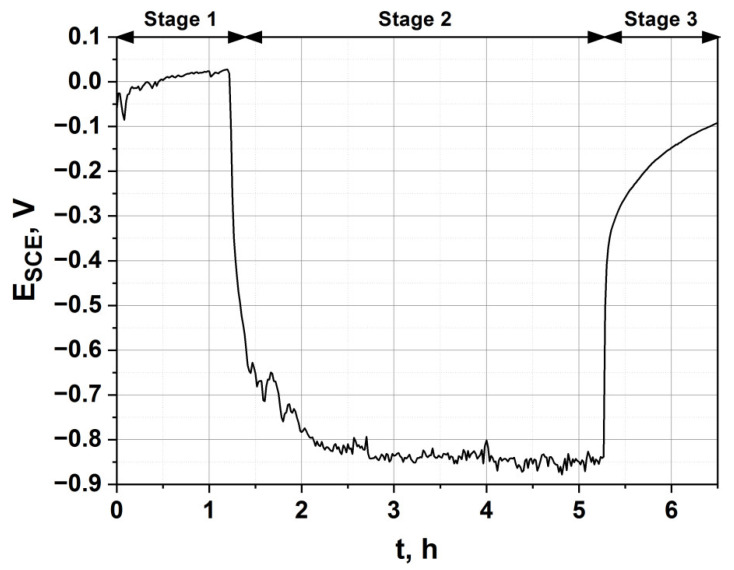
Change in the corrosion potential during the tribological test in Ringer’s solution at 25 °C of the alloy hardened at 700 °C.

To investigate the passivation kinetics of the hardened alloy in a condition where the oxygen-rich hardened layer of the alloy is abraded, tribological tests were performed in the presence of Ringer’s solution. The tests were coupled with simultaneous measurement of the OCP (Figure 2). Figure 11 shows the change in the corrosion potential over time. In the diagram, we can distinguish 3 characteristic stages:

i) Stage 1—increase and stabilization of the stationary potential;
ii) Stage 2—a drop of the corrosion potential, resulting from the wear of the alloy’s surface, with the assumed sliding distance equaling 1000 m;
iii) Stage 3—an increase of the stationary potential after the interruption of the friction process of the alloy’s surface.

The friction process was activated after 1 h 20 min of stabilization in Ringer’s solution. A significant drop in the potential corrosion value was observed, resulting from the change in the measurement conditions. As a result of friction, a groove was formed, with significant surface roughness and less passivating, thereby showing a considerable drop of the corrosion potential. The potential reached about −846 ± 50 mV vs. SCE and was maintained at this value during the whole friction process. When the friction process was terminated (Stage 3), an increase in the corrosion potential was observed. The increase in the potential took place abruptly. The strengthened outer layer of the alloy treated at 700 °C provided good protection against tribological wear in a corrosive environment, and the passive film was quickly restored.

## 4. Conclusions

In this work, the oxygen hardening of Ti–13Nb–13Zr alloy by plasma glow discharge at 700–1000 °C was studied.

1. The hardening temperature had a significant influence on the alloy microstructure and thickness of the hardened zone. A refinement of the α’ laths of the near β-phase Ti alloy was observed. The thickness of the hardened zone and grain size of the bulk alloy both increased with increasing temperature. In addition, voids and microcracks were observed in the near-surface zone of the alloy treated at 1000 °C;
2. The outer hardened zone consisted mainly of the Ti α’ (O) solid solution with small amounts of fine laths of the Ti α’’ (O) solid solution in the β phase. Oxygen enrichment in a depth of up to 6 μm was found;
3. The oxidation of the Ti–13Nb–13Zr alloy under glow discharge conditions resulted in a significant increase of hardness and elastic modulus compared with the base alloy. The best results were found for the alloy hardened at 700 °C. With an increasing temperature, a decrease in both hardness and modulus of elasticity in the hardened zone were observed;
4. The hardened titanium alloy zone significantly reduces abrasive wear, and the wear resistance is proportional to the hardness of the alloy;
5. Oxygen hardened alloy does not adversely affect the corrosion resistance;
6. The friction reduces the corrosion resistance of the oxygen-hardened Ti–13Nb–13Zr alloy. However, when the friction process was stopped, the corrosion potential was quickly restored. The strengthened outer layer of the alloy treated at 700 °C provides good protection against tribological wear in a corrosive environment.

## Figures and Tables

**Table 1:** Microhardness (H_IT_), elastic modulus (E_IT_), the penetration depth of the indenter (h_max_), and wear rate (W_v_) of the Ti–13Nb–13Zr alloy.

Sample of Ti–13Nb–13Zr Alloy	h_max_ (nm)	H_IT_ (GPa)	E_IT_ (GPa)	W_v_·10^−6^ (mm^3^/Nm)
Hardened at 700 °C	880 ± 37	12.8 ± 0.8	180 ± 16	2.3 ± 0.3
Hardened at 850 °C	925 ± 52	9.8 ± 0.9	175 ± 20	3.1 ± 0.3
Hardened at 1000 °C	1407 ± 131	6.9 ± 0.7	152 ± 17	5.8 ± 0.7
As-received	1662 ± 82	3.9 ± 0.2	79 ± 9	1250 ± 46

**Table 2:** Chi-squared (χ^2^) values obtained by fitting equivalent electrical circuit with Z View software and electrochemical parameters for the as-received and treated titanium alloy.

Sample	χ^2^	R_1_ (Ω * cm^2^)	CPE-T (Fs^n−1^ cm^−2^)	CPE-P	R_2_ (Ω * cm^2^)
As-received alloy	0.005	12.30 ± 0.12	4.31 × 10^−5^ ± 0.03 × 10^−5^	8.96 × 10^−1^ ± 0.02 × 10^−1^	3.22 × 10^5^ ± 0.06 × 10^5^
Alloy hardened at 700 °C	0.004	41.99 ± 0.25	4.35 × 10^−5^ ± 0.02 × 10^−5^	8.70 × 10^−1^ ± 0.01 × 10^−1^	7.26 × 10^5^ ± 0.11 × 10^5^

## Data Availability

The data presented in this study are available on request from the corresponding author.
